# Therapeutic Potential of Polyphenols in Amyotrophic Lateral Sclerosis and Frontotemporal Dementia

**DOI:** 10.3390/antiox10081328

**Published:** 2021-08-23

**Authors:** Valentina Novak, Boris Rogelj, Vera Župunski

**Affiliations:** 1 Chair of Biochemistry, Faculty of Chemistry and Chemical Technology, University of Ljubljana, SI-1000 Ljubljana, Slovenia; vn4556@student.uni-lj.si (V.N.); boris.rogelj@ijs.si (B.R.); 2 Department of Biotechnology, Jozef Stefan Institute, SI-1000 Ljubljana, Slovenia

**Keywords:** ALS, FTD, polyphenols, neurodegeneration, resveratrol, curcumin, catechin, EGCG

## Abstract

Amyotrophic lateral sclerosis (ALS) and frontotemporal dementia (FTD) are severe neurodegenerative disorders that belong to a common disease spectrum. The molecular and cellular aetiology of the spectrum is a highly complex encompassing dysfunction in many processes, including mitochondrial dysfunction and oxidative stress. There is a paucity of treatment options aside from therapies with subtle effects on the post diagnostic lifespan and symptom management. This presents great interest and necessity for the discovery and development of new compounds and therapies with beneficial effects on the disease. Polyphenols are secondary metabolites found in plant-based foods and are well known for their antioxidant activity. Recent research suggests that they also have a diverse array of neuroprotective functions that could lead to better treatments for neurodegenerative diseases. We present an overview of the effects of various polyphenols in cell line and animal models of ALS/FTD. Furthermore, possible mechanisms behind actions of the most researched compounds (resveratrol, curcumin and green tea catechins) are discussed.

## 1. Introduction

With the ageing population, the treatment and management of neurodegenerative diseases is a major and increasing challenge for health care systems and societies around the world [1]. Amyotrophic lateral sclerosis (ALS) is a neurodegenerative disease that affects motor neurons, resulting in deterioration of motor function, and frontotemporal dementia (FTD) is a neurodegenerative disorder characterised by changes in personality, behaviour, and language. The development of both diseases is a progressive and ultimately fatal multistep process with a complex genetic and molecular background. Despite extensive research efforts, only two treatment options with limited effects on survival and motor function are currently approved for ALS. The vast majority of compounds researched as possible ALS therapies until today were found to be ineffective in clinical trials, highlighting the need for further research [2]. Currently, only symptomatic treatments with limited effects are available for FTD [3].

Polyphenols are natural compounds whose neuroprotective effects have been demonstrated in various models of neurodegenerative diseases such as Alzheimer’s and Parkinson’s disease. These compounds are being explored for possible dietary intervention and supplementation as preventive measures against neurodegenerative diseases, and also as possible candidates for therapies to slow disease progression and alleviate symptoms [4]. Due to the lack of disease-changing treatments for ALS/FTD and the growing interest in natural compounds as therapeutic agents, this article reviews an intriguing topic of potential use of polyphenols in the development of treatments for ALS/FTD symptoms.

### 1.1. Amyotrophic Lateral Sclerosis and Frontotemporal Dementia

ALS is a neurodegenerative disease characterised by progressive loss of both upper and lower motor neurons. Initial signs of the disease may include weakness of the limbs (in spinal-onset ALS) or difficulties with speech and swallowing (in bulbar-onset ALS) [5]. Disease progression eventually leads to paralysis and death from respiratory failure, on average 24 to 50 months after onset [6,7,8,9,10]. The worldwide incidence of ALS is 1.75 with a reported mean age at diagnosis between 51 and 69 years [11,12]. ALS cases can be divided into the familial form of the disease (fALS, 5–15% of patients), where there is a clear family history, and the predominant sporadic form (sALS) [13]. Frontotemporal dementia (FTD) is a type of dementia primarily associated with alterations in the frontal and temporal lobes. Symptoms manifest as changes in behaviour, personality, language, and motor skills [14,15]. The incidence of FTD is 1.6 and the mean age of onset is 65 years [16]. FTD can be divided into one behavioural (bvFTD) and two language variants (or primary progressive aphasias (PPA)) [14]. Mean survival time for most forms of FTD is approximately 8 years [17]. Up to 40% of FTD patients have a family history of the disease [18,19].

**Figure 1:**
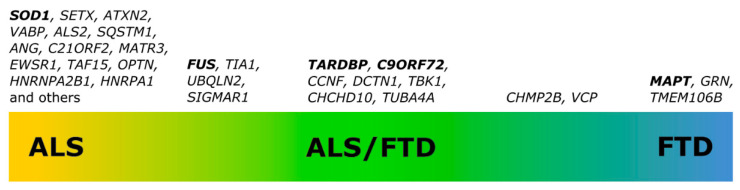
Genes involved in pathologies along the ALS-FTD spectrum. The most common genetic causes of the disease are highlighted in bold.

Clinical, genetic, pathological and biochemical data show that there is an overlap between ALS and FTD. First observations that ALS and FTD might be connected were made in the early 1990s [20,21]. Data show that about half of ALS patients have cognitive impairment and 15% meet the criteria for FTD [22,23]. Similarly, about 30% of patients with FTD develop signs of motor dysfunction and 10–15% have ALS [24,25]. The discovery of common genetic causes and biological mechanisms further confirmed that ALS and FTD are closely associated (Figure 1) [5,26].

ALS and FTD pathologies are multistep processes that affect many aspects of cellular activity. The most prominent pathological hallmark of both ALS and FTD are changes in protein homeostasis, including protein misfolding and aggregation, altered localisation, and defects in autophagic and proteasomal degradation. The combination of these mechanisms leads to the formation of toxic cytoplasmic inclusions in motor neurons and surrounding cells. Proteins that predominantly form these structures are two RNA-binding proteins, TAR DNA binding protein (TDP-43, protein product of *TARDBP*), and fused in sarcoma (FUS), microtubule-associated protein tau (gene *MAPT*), and superoxide dismutase 1 (SOD1) [27,28]. The correlation between pathology and genetics is complex [29,30,31]. Pathologically, 97% of ALS cases have pathognomonic TDP-43 aggregates, while only 1% of those are associated with mutations in TDP-43 and in the rest TDP-43 is not mutated. A total of 1% of ALS shows FUS aggregates, all of which are associated with mutations in FUS. Mutations in FUS or TDP-43 are extremely rare in FTD; however, 50% of FTD have TDP-43 aggregates and 10% of FTD have FUS aggregates. A total of 40% of FTD is tau aggregates. Impairments in protein turnover and clearance are also observed. Mutations in genes associated with different stages of autophagy are also causative for ALS/FTD, from autophagy regulating activities of C9ORF72 to impaired functions of autophagic receptors SQSTM1 and optineurin [32,33,34,35,36,37].

In healthy cells, TDP-43 and FUS are predominantly nuclear RNA/DNA-binding proteins with functions in RNA splicing, transcription, microRNA biogenesis, and mRNA transport [38,39,40,41,42,43,44,45,46,47]. Both play important parts in ribonucleoprotein coacervates that form membrane-less organelles such as stress granules in the cytoplasm and paraspeckles in the nucleus [48,49]. In ALS/FTD, FUS or TDP-43 mislocalise to the cytoplasm and form aggregates that are most likely toxic, although loss of function from the nucleus may also be the key disease-causing factor. This mislocalisation is instigated by a number of disruptions, including dysfunctions in proteostasis, nucleocytoplasmic shuttling, and the cellular stress response [50,51,52]. Upon stress, TDP-43, FUS, and some other ALS-associated RNA-binding proteins separate into stress granules, which may be the first step in the formation of insoluble aggregates [53,54]. Another common factor in the disruption of RNA metabolism is G4C2 repeat expansions in the C9ORF72 gene, which are the most common cause of familiar forms of ALS/FTD [55,56,57]. The repeats form stable nucleic acid secondary structures known as G-quadruplexes, hairpin loops, and i-motifs, that sequester RNA-binding proteins and form nuclear foci similar to paraspeckles, or can be translated into toxic dipeptide repeats via repeat-associated non-ATG translation [58,59,60,61,62,63,64,65].

Mitochondria play a central role in neurons, primarily fulfilling high needs for energy. ALS/FTD-associated changes include defects in oxidative phosphorylation and calcium homeostasis, elevated production of ROS, structural impairments, and reduced clearance of damaged mitochondria [66]. Changes in mitochondrial morphology are observed in cells overexpressing mutant SOD1, FUS, or TDP-43 [67,68,69,70]. The increased localisation of mutant SOD1 in the mitochondrial intermembrane space causes mitochondrial dysfunction and toxicity to neurons [71,72,73]. Overall, mitochondrial changes result in decreased electron transport chain activity and reduced ATP production [66]. Moreover, oxidative stress has been proposed to be crucial in ALS pathogenesis and has been well documented in patient samples [74,75,76].

### 1.2. Currently Used Therapies for ALS/FTD

Treatments currently in clinical trials for ALS/FTD were comprehensively reviewed by Liscic et al. [26]. Therapeutic targets include a reduction in glutamate excitotoxicity and protein aggregation, upregulation of certain heat shock proteins, and activation of troponin in skeletal muscle. Interesting novel strategies for ALS/FTD treatment may also come from stem cell therapy, non-invasive brain stimulation, and the growing knowledge of the influence of the gut microbiota on the development of neurological diseases [26]. Currently, only two drugs are approved for the treatment of ALS. Riluzole was approved for clinical use in 1995 and trials observed reduced one year mortality and slower deterioration of muscle function [26,77,78]. The mechanisms behind the beneficial effects of riluzole are not entirely clear. Different neuroprotective actions have been proposed, such as inhibition of glutamate excitotoxicity, blockade of Ca^2+^- or Na^+^-ion channels, and modulation of GABA pathways [79]. In recent years, some countries have also approved the use of edaravone (also known as MCI-186 or Radicava) for the treatment of ALS [26]. Its actions could benefit a subgroup of patients with early onset and rapidly progressive disease [80]. Edaravone is thought to act as an antioxidant and free radical scavenger, but the mechanisms are not well understood [81]. There are currently no approved direct treatments for FTD, other than symptom management [82].

## 2. Therapeutic Potentials of Polyphenols in ALS/FTD

**Figure 2:**
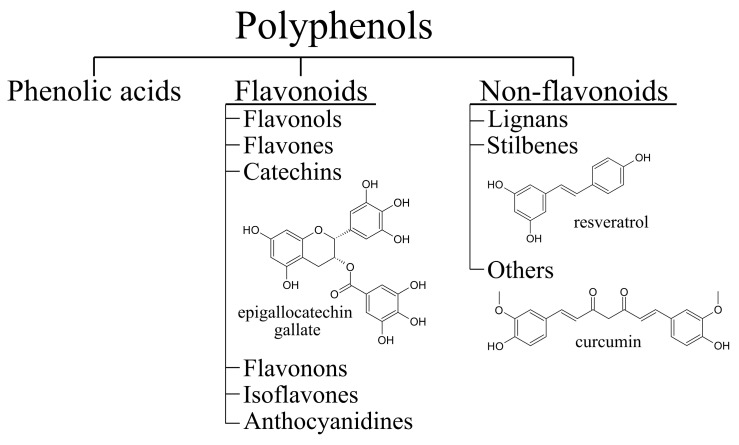
Classification of polyphenols with structural formulas of epigallocatechin gallate (EGCG), resveratrol and curcumin.

Many potential therapeutic compounds have antioxidant and anti-inflammatory properties. Polyphenols (Figure 2) are a diverse group of naturally occurring compounds with a characteristic chemical structure that has one or more phenolic rings. They are found in plant foods such as fruits, vegetables, and whole grains [83,84]. In plants, polyphenols are categorised as secondary metabolites and have functions in normal growth as well as in the plant defense system [85]. They are synthesised in the shikimate and phenylpropanoid pathways [86]. Many different polyphenols have been described to have neuroprotective effects in mammalian cell and animal models of ALS/FTD [87]. In this review, the focus will be on resveratrol, epigallocatechin gallate (EGCG), and curcumin (Figure 2). We will also explore the effects of some other flavonoids and phenolic acids in the context of ALS/FTD.

### 2.1. Resveratrol

Resveratrol (3,5,4′-trihydroxystilbene) is a polyphenol found in grapes, red wine, berries, and peanuts [88]. Both cis- and trans- isomers occur naturally, with trans-form being the focus in terms of potential neuroprotective activity [89]. Effects of resveratrol in ALS were first demonstrated in neuronal cell lines expressing the SOD1^G93A^ mutant [90,91,92]. Resveratrol treatment halved the cell death observed as a consequence of SOD1-mediated toxicity [90]. Treatments of mouse motor neuron cells NSC34 expressing SOD1^G93A^ showed a minor dose-dependent improvement in cell viability and a simultaneous reduction in the concentration of cytosolic ROS [91]. Administration of resveratrol protected rat cortical motor neurons from the toxic effects of cerebrospinal fluid (CSF) from ALS patients [93]. Further studies in mice ALS models expressing mutant SOD1^G93A^ showed conflicting results, which are probably a consequence of different protocols on dosing and route of administration. Chronic oral administration of resveratrol at 25 mg/kg/day did not improve motor abilities and life span of ALS mice [94]. On the other hand, intraperitoneal injections of 20 mg/kg/twice a week improved survival and delayed the onset of ALS [95]. A similar positive effect on survival and motor function was observed with a higher dose (160 mg/kg/day) administered orally [96]. The neuroprotective effects of resveratrol in ALS mice have been further demonstrated in coadministration with other potential therapeutics [97,98]. Resveratrol has also been researched in models of tauopathies, a hallmark of FTD, but the overall effects on tau aggregation are inconclusive [99].

The predominant mechanism behind the neuroprotective effect of resveratrol is the activation of SIRT1, a NAD^+^-dependent protein deacetylase [90,92,95,96,100]. Structural studies suggested a mechanism in which resveratrol acts as an adaptor for the interaction between the peptide substrate and SIRT1 [101]. Many downstream mechanisms of SIRT1 targets have been proposed as possible mediators of the beneficial effects. SIRT1 deacetylates p53 [90,96], a known tumor suppressor protein involved in mechanisms of motor neuron cell death [102]. Resveratrol treatment upregulates factors involved in mitochondrial biogenesis, which could improve altered energy metabolism observed in ALS [92,96]. SIRT1 also targets HSF1 (heat shock factor 1) that activates several heat shock proteins. Their activity as chaperones possibly mitigates formation of toxic protein aggregates [95]. Normalisation of autophagic flux was also observed in resveratrol-treated ALS mice, but it is not clear whether SIRT1 is involved [96]. Independent of SIRT1, resveratrol can also activate AMPK (AMP-activated protein kinase) [96,103] that has downstream targets involved in neuroprotective mechanisms [104]. Moreover, a molecular mechanistic study on SOD1^G93A^ showed a stabilising effect of resveratrol that could impede the aggregation of mutant protein [105]. A similar inhibitory effect was observed in aggregation studies of wt SOD1 [106].

### 2.2. Curcumin

Curcumin (diferuloylmethane) is the predominant curcuminoid found in turmeric (*Curcuma longa*), which is widely used in traditional Indian medicine. The potential benefits of curcumin are being explored in many neurodegenerative diseases. In models of Alzheimer’s and Parkinson’s disease, curcumin can reduce oxidative stress, affect toxic protein aggregation, and protect against apoptosis [107,108].

Regarding ALS, curcumin was shown to impede aggregation of reduced wt SOD1 in vitro by binding its aggregation prone regions. Curcumin-bound SOD1 aggregates were smaller, unstructured, and less cytotoxic [109]. A similar effect of inhibiting beta-sheet formation and aggregation was observed with tau, a protein involved in FTD [110]. In contrast, the binding of curcumin to tau aggregates was not observed in post-mortem brain tissue sections from FTD patients [111].

Curcumin presents a challenge for in vivo use due to its poor absorption, fast metabolism, and rapid elimination. Several strategies can be utilised to overcome the low oral bioavailability of curcumin [112]. The protective effect of an analogue, dimethoxy curcumin, was demonstrated in a neuronal cell line expressing TDP-43 mutants Q331K or M337V. Dimethoxy curcumin restored mitochondrial damage by improving transmembrane potential, increasing electron transfer chain complex I activity, and upregulating UCP2 (uncoupling protein 2) [113]. The same compound also improved abnormally high excitability of cells expressing mutant TDP-43 [114]. Furthermore, an improved curcumin analogue, monocarbonyl dimethoxycurcumin C, prevented aggregation of mutant TDP-43 and reduced oxidative stress, possibly due to increased expression of heme oxygenase-1 [115].

Another approach to improve the bioavailability of curcumin is delivery using nanoparticles. The potential for ALS treatment was demonstrated with curcumin-loaded inulin-d-alfa-tocopherol succinate micelles, which were effectively delivered into mesenchymal stromal cells [116]. Furthermore, the efficiency of a turmeric supplement in nanomicelles was tested in a clinical trial involving 54 ALS patients treated primarily with riluzole. Nanocurcumin improved the survival probability of the patients, but did not significantly improve their motor function [117].

### 2.3. Catechins

Green tea, produced from the leaves and buds of *Camellia sinensis*, is rich in polyphenols catechins, predominantly (−)epigallocatechin gallate (EGCG), but also (−)-epigallocatechin (EGC), (−)-epicatechin gallate (ECG), (−)-epicatechin (EC), and (+)-catechin [118]. In ALS models, EGCG has been shown to protect motor neuron cells from oxidative stress and mitochondrial damage [119]. Presymptomatic oral supplementation of EGCG at doses of at least 2.9 mg EGCG/kg body weight in SOD1^G93A^ mice significantly delayed symptom onset, improved motor function, and increased lifespan [120,121].

EGCG likely acts by upregulating a prosurvival signaling pathway PI3K/Akt. Among other pathways, PI3K/Akt regulates the activity of GSK-3. Increased GSK-3 levels are associated with the formation of neurofibrillary tangles and neuronal death. In addition, GSK-3 induces apoptosis through downstream signaling, including mitochondrial damage and caspase-3 activation. It was shown that Akt phosphorylates GSK-3, resulting in less mitochondrial damage [119]. Observations in ALS mice further confirm an increase in PI3K/Akt and a decrease in death signals such as caspase-3, cytosolic cytochrome c, and cleaved PARP (poly (ADP-ribose) polymerase) [120]. EGCG also has antioxidant and anti-inflammatory effects on microglia and astrocytes [121]. In addition, it can decrease lipid peroxidation, but has no effect on iron metabolism despite its presumed chelating abilities [122]. A molecular docking study showed the potential of EGCG to reduce mutant SOD1 aggregates [123]. In vitro studies confirmed an inhibitory effect on apo-SOD1 aggregation [124]. It has also been shown that the addition of EGCG induces oligomerisation of TDP-43 and inhibits its degradation into toxic aggregation-prone fragments [125]. In FTD, inhibition of tau filament formation was observed for ECG, but not for EC [126].

### 2.4. Other Flavonoids

In addition to green tea catechins, several other flavonoids have been tested in ALS/FTD models. Presymptomatic administration of 2 mg/kg body weight of an anthocyanin-enriched strawberry extract with callistephin (pelargonidin 3-glucoside) as the predominant component delayed ALS onset, preserved grip strength, and prolonged survival in SOD1^G93A^ mice [127]. Oral supplementation of fisetin (3,3,4,7-tetrahydroxyflavone) improved motor functions, delayed disease onset, and increased survival in SOD1^G93A^ mice (at a dosage of 9 mg/kg) and SOD1^G85R^ *Drosophila melanogaster*. The predominant mechanism behind the activity of fisetin in motor neuron cell lines expressing SOD1^G93A^ appears to be the activation of the ERK pathway involved in the regulation of cell survival. Moreover, fisetin decreased both wt and mutant SOD1 levels in cells, possibly by activating autophagy [128].

A computational study confirmed the binding of kaempferol (3,4′,5,7-tetrahydroxyflavone) and kaempferide to mutant SOD1^G85R^ [129]. Both compounds were experimentally shown to have antioxidant properties and could reduce the formation of SOD1^G85R^ aggregates in N2a mouse neuroblastoma cells. Kaempferol could act via increased phosphorylation of AMPK and downstream induction of autophagy [130]. The antioxidant effect of quercetin (3,3′,4′,5,7-pentahyroxyflavone) was first observed in lymphoblast cell lines from ALS patients [131]. In vitro tests showed that quercetin glycosides, namely quercitrin and quercetin 3-beta-d-glucoside, inhibit misfolding and aggregation of SOD1^A4V^ mutant [132]. A similar effect on aggregation was observed with quercetin and baicalein [133]. Furthermore, preventive administration of quercetin in rats reduced oxidative stress, defective mitochondria, and brain cell death caused by aluminium exposure [134].

SOD1^G93A^ mice treated with 5 mg/kg 7,8-dihydroxyflavone exhibited significantly improved motor performance and increased numbers of spinal motor neurons compared with untreated animals [135]. Interestingly, it was observed that treatment with 16 mg/kg genistein (4′,5,7-trihydroxyisoflavone) had a protective effect on disease progression in male SOD1^G93A^ mice [136]. In contrast, in further studies, a delay in symptoms and higher survival of motor neurons was observed in both sexes, possibly due to anti-inflammatory effects and restored autophagy [137]. Twice-daily administration of 700 mg luteolin (3′,4′,5,7-tetrahydroxyflavone) in combination with palmitoylethanolamide showed some improvement of symptoms in patients with FTD [138].

### 2.5. Phenolic Acids and Derivatives

Phenolic acids are found in fruits, coffee, tea, and grains. Their diverse neuroprotective effects make them interesting candidates for better ALS therapies. It has been reported that protocatechuic acid administration at 100 mg/kg in SOD1^G93A^ mice prolongs survival, improves motor function, and reduces gliosis [139]. Caffeic acid phenethyl ester (CAPE) showed a dose-dependent improvement in survival and a simultaneous reduction in cytosolic ROS in the NCS34 cell line expressing SOD1^G93A^. CAPE decreased the activation of the oxidative stress-associated transcription factor NF-κB and activated the antioxidant response element (ARE) [91]. Further studies in SOD1^G93A^ mice confirmed that daily administration of 10 mg/kg CAPE after disease onset slowed symptom progression and prolonged survival. A reduction in glial activation and phospho-p38 levels was observed as a result [140]. Gallic acid and wedelolactone improved locomotor function and motor learning abilities in an aluminium or quinolinic acid-induced rat model of sALS. The effects may be due to a reduction in inflammatory cytokines, normalisation of L-glutamate levels, and decreased activation of caspase-3 [141,142]. Rosmarinic acid, the main compound in rosemary (*Rosmarinus officinalis*) extract, reduced weight loss, improved motor performance, and prolonged survival of SOD1^G93A^ mice [143,144]. The effects of treatment with higher doses were compared with the established ALS therapeutic agent riluzole, but were not found to be more effective [144].

### 2.6. Overview of Potential Therapeutic Effects of Polyphenols in ALS and FTD

We have summarised the therapeutic implications of polyphenols, including their proposed mechanisms in animal and cell line models of ALS and FTD (Table 1). The predominant mechanism behind the neuroprotective role of resveratrol is the activation of SIRT1. Its downstream targets may impact processes such as neuronal survival, mitochondrial biogenesis, and prevention of protein aggregate formation, all of which contribute to the observed delay in symptoms and increased viability in ALS models [90,92,95,96]. Curcumin derivatives show neuroprotective value through several mechanisms, such as restoring mitochondrial functions, normalising cell excitability, and preventing the formation of toxic protein aggregates [113,114,115]. Green tea catechin EGCG has been observed to upregulate a prosurvival signaling pathway PI3K/Akt and decrease signals leading to cell death, such as activation of caspase-3, which is associated with apoptosis [119,120]. Both resulted in the delayed onset of ALS and increased survival in mice models treated with EGCG [120,121]. Fisetin acts by activating the ERK pathway, which modulates cell survival and upregulates HO-1, both of which contribute to the cellular response against oxidative stress [128]. Another mechanism exerted by some polyphenols is the downregulation of the NF-κB pathway that, overall, has an anti-inflammatory effect [91].

The importance of the gut–brain axis in ALS/FTD has been recognised. On the one hand, polyphenols may serve as prebiotics and alter the gut microbiota, affecting disease pathogenesis [146], (for a detailed review, see [147]). On the other hand, certain polyphenols such as EGCG are degraded by some gut microbiota, which reduces their bioavailability [148,149]. However, some metabolites do target the brain and have beneficial effects on neurons [118,150].

## 3. Conclusions

Polyphenols offer new possibilities for the development of therapies for ALS/FTD. However, more research is needed in this field, including strategies for effective targeting and delivery to the site of action. When evaluating the therapeutic potential of polyphenols, we must also consider their uptake in the gut, degradation by the microbiota, and the delivery to the brain. Therefore, it is important whether polyphenols are consumed or administered intravenously and how well they can cross the blood–brain barrier [151,152]. Another hurdle for potential ALS/FTD medication is translating findings from animal models into successful clinical trials. Additional aspect of potential variability in successful treatment lies in the use of purified polyphenols or plant extracts that may act synergistically. Most of the findings reviewed here come from various successful preclinical stages and have yet to be tested in humans. Nevertheless, polyphenols have the potential to improve the treatment of ALS/FTD, either through the development of new drugs or as dietary supplements.

## Figures and Tables

**Table 1:** Therapeutic implications of different polyphenols in ALS and FTD models.

Compound	Animal/Cell Line	Mechanism of Action	Outcome	Ref.
resveratrol	rat cortical primary neurons expressing SOD1^G93A^	activation of SIRT1	reduced cell death	[90]
	NCS34 cell line expressing SOD1^G93A^	antioxidant activity	reduction in ROS, increased viability	[91]
	VSC4.1 cell line expressing hSOD1^G93A^	activation of SIRT1, mitochondrial biogenesis	increased viability, reduced apoptosis	[92]
	rat cortical neurons with ALS-patient CSF	possibly reduction in cytosolic Ca^2+^ concentration	increased viability	[93]
	mice expressing SOD1^G93A^	activation of SIRT1, heat shock protein response	delayed onset, increased survival	[95]
	mice expressing SOD1^G93A^	activation of SIRT1, mitochondrial biogenesis, normalised autophagic flux	delayed onset, improved motor function, increased survival	[96]
	bone marrow-mesenchymal stem cells of ALS patients	activation of SIRT1 and AMPK	increased differentiation rate	[103]
dimethoxy curcumin	NSC34 cell line expressing TDP-43^Q331K^, TDP-43^M337V^	decreased expression of UCP2, improved mitochondrial transmembrane potential and morphology	improved mitochondrial function	[113]
	NSC34 cell line expressing TDP-43^Q331K^	not determined	lowered excitability, no observed change in survival	[114]
monocarbonyl dimethoxycurcumin	NSC34 cell line expressing TDP-43^Q331K^	upregulation of HO-1	reduced oxidative stress and toxicity	[115]
epigallocatechin gallate	VSC4.1 cell line expressing SOD1^G93A^	protection from oxidative stress, increase in survival signals through PI3K	increased viability, reduced apoptosis	[119]
	mice expressing SOD1^G93A^	increase in survival signals through PI3K	delayed onset, increased lifespan	[120]
	mice expressing SOD1^G93A^	reduced activation of NF-κB and caspase-3	delayed onset, increased lifespan	[121]
	rat spinal cord culture with THA (induced glutamate excitotoxicity)	decrease of lipid peroxidation	increased viability	[122]
anthocyanin enriched strawberry extract	mice expressing SOD1^G93A^	preservation of neuromuscular junctions, reduction in reactive astrocytes	delayed onset, increased survival	[127]
fisetin	NCS34 cell line expressing SOD1^G93A^	antioxidant activity, activation of ERK pathway	increased viability	[128]
	*Drosophila melanogaster* expressing SOD1^G85R^	antioxidant activity, activation of ERK pathway	increased survival, improved motor function	[128]
	mice expressing SOD1^G93A^	antioxidant activity	delayed onset, increased survival, improved motor function	[128]
kaempferol	N2a cells expressing SOD1^G85R^	reduction in mutant SOD1 aggregates, induction of autophagy (AMPK)	increased viability	[130]
quercetin	lymphoblast cell lines from ALS patients	reduction in ROS	not determined	[131]
	rats, aluminium-induced neurodegeneration	reduced oxidative stress, improved mitochondrial function	increased neuronal viability, inhibition of apoptosis	[134]
7,8-dihydroxyflavone	mice expressing SOD1^G93A^	not determined, possibly as TrkB agonist	improved motor function, higher motor neuron count and density	[135]
genistein	mice expressing SOD1^G93A^	not determined	delayed onset and increased survival in males	[136]
	mice expressing SOD1^G93A^	anti-inflammatory, autophagy promotion	delayed onset and improved motor performance, increased survival in both sexes	[137]
protocatechuic acid	mice expressing SOD1^G93A^	anti-inflammatory, preservation of neuromuscular junctions	increased survival, improved motor performance	[139]
caffeic acid phenethyl ester	NCS34 cell line expressing SOD1^G93A^	reduced activation of NF-κB, activation of antioxidant response element	increased viability, reduction in ROS	[91]
	mice expressing SOD1^G93A^	anti-inflammatory, anti-cell death signals	slower progression, increased survival	[140]
gallic acid	rats, aluminium- or quinolinic acid-induced neurodegeneration	antioxidant and anti-inflammatory activity, prevention of apoptosis, reduction in glutamate	improved motor function	[141,142]
rosmarinic acid	mice expressing SOD1^G93A^	not determined	increased survival, improved motor function, reduced weight loss	[143]
	mice expressing SOD1^G93A^	antioxidant activity	increased survival, improved motor function	[144]
nordihydroguaiaretic acid	mice expressing SOD1^G93A^	TNFα antagonist	increased survival, reduced weight loss	[145]
